# Correlates of Adult Binge Drinking: Evidence from a British Cohort

**DOI:** 10.1371/journal.pone.0078838

**Published:** 2013-11-13

**Authors:** Helen Cheng, Adrian Furnham

**Affiliations:** 1 Department of Psychology, University College London, London, United Kingdom; 2 BI: Norwegian Business School, Oslo, Norway; University Of São Paulo, Brazil

## Abstract

**Objective:**

To investigate whether parental social class and cognitive ability in childhood, as well as social and psychological factors, particularly personality traits, are independently associated with binge drinking in 50 year old adults assessed in a longitudinal birth cohort study.

**Method:**

17,415 babies born in Great Britain in 1958 and followed up at 11, 33, and 50 years of age. Their binge drinking alcohol abuse at aged 50 was the outcome measure.

**Results:**

6,478 participants with data on parental social class, childhood cognitive ability, educational qualifications at age 33, personality traits, psychological distress, occupational levels, and alcohol consumption (all measured at age 50) were included in the study. Using logistic regression analyses, results showed that parental social class, childhood intelligence, educational qualifications, occupational levels, personality traits (Extraversion and Disagreeableness), as well as psychological distress, were all significantly and independently associated with adult excessive alcohol use. Men tended to binge drink more than women (22% in men and 9.8% in women).

**Conclusion:**

Both social and psychological factors influence adult excessive alcohol consumption. Personality traits play a more important role than previously understood. There appears to be a distinction between the frequency and dose level of alcohol consumption.

## Introduction

The prevalence of alcohol misuse has increasingly become one of salient social concern. It not only affects individuals’ daily functioning and health [1], but also has costly social and societal consequences. Previous studies have shown that excessive alcohol use is related to various social, economic, and psychological factors. Among the social factors, parental social class, education, and occupational levels are found to be related to alcohol use and misuse [2].

This study focuses on a set of demographic (gender, education), personality (the Big-Five), psychological (cognitive ability, psychological distress), and sociological (social class) factors, and sets out to investigate the associations between these factors and adult binge drinking, a topic which has attracted considerable research interests in the past decade [3]–[7].

There is evidence that childhood cognitive ability is positively related to a higher average intake of alcohol and to drinking more frequently [5]–[7], yet various studies have shown that intelligence measured in childhood has a positive association with health and mortality [8]–[11]. It is plausible though to assume that people with higher intelligence are less likely to engage in hazardous behaviours, such as binge drinking, which may potentially impair one’s mental and physical health [1], [3].

In a recent study, a research team [1] conducted a meta-analysis based on 84 studies extracted from over 4000 studies, with inclusion criteria, on the associations between alcohol consumption and a number of cardiovascular disease outcomes. Results revealed that the pooled adjusted relative risks for alcohol drinkers, relative to non-drinkers in random effects models, were 0.75 for cardiovascular disease mortality, 0.71 for incident coronary heart disease, and 0.75 for coronary heart disease. The same study also reported that dose-response analysis revealed that modest alcohol intake (1–2 drinks a day) compared with no alcohol use was associated with a reduced risk of multiple cardiovascular outcomes [1].

Associations between personality, health, and longevity are increasingly documented, though the causal directions are less clear [12]. Personality is found to be related to various health-related behaviours [13], such as alcohol-related risky behaviours [14], and longevity [15], [16].

There have been various studies on personality correlates of alcohol consumption. In one study, 900 British adults were tested and it was found that alcohol consumption was positively correlated with sociability and trait Extraversion, and negatively with Conscientiousness and willingness to conform [17]. In another study, 198 teenagers were examined and it was found that Extraversion and Psychoticism positively correlated with the quantity and frequency of alcohol consumption [18]. In a study of 463 young Americans, a research team [19] found impulsivity, risk taking, and excitement seeking were related to both alcohol use and problems. Many of these studies were cross-sectional, and did not have measures of the “Big Five” personality traits, currently agreed as the most comprehensive and accepted model of personality [20]. Further, they were unable to take into account other possibly confounding demographic and sociological factors, like social class and education.

The links between mental health, especially anxiety disorders, and alcoholism are well documented [11]–[23]. For example, in a clinical sample [24] it was found that forty per cent of a sample of 75 inpatient alcoholics received a lifetime diagnosis of one or more anxiety disorders. Similar findings were found in community samples [23].

This study has many advantages over previous studies: it uses a large, representative, longitudinal sample followed over 50 years. Further, because we had demographic, psychological, *and* sociological variables, we were able to ascertain the relative power of variables, especially personality traits, in predicting binge drinking. This study set out to investigate whether parental social class and cognitive ability in childhood, and adult social and psychological factors are independently associated with excessive alcohol consumption in 50 year old adults in a nationally representative, longitudinal birth cohort study.

Based on previous studies, it is hypothesised that: 1) Childhood factors, parental social class, and intelligence would be significantly associated with adult binge alcohol use: the higher scores of childhood cognitive ability the lower dose level of alcohol intake; 2) Educational and occupational levels would be significantly associated with adult binge alcohol use; 3) Personality traits, particularly Extraversion and Conscientiousness, and mental distress would be significantly associated with adult binge alcohol use; 4) Parental social class and childhood intelligence, educational and occupational levels, traits Extraversion and Conscientiousness, and psychological distress would be independently associated with the outcome variable.

## Method

### Sample

The National Child Development Study 1958 is a large-scale longitudinal study of the 17,415 individuals who were born in Great Britain in a week in March 1958 [24]. The following analysis is based on data collected when the study participants were at birth, at ages 11, 33 and at 50 years. 14,134 children at age 11 completed tests of cognitive ability (response = 87%). At age 50, 8,532 participants completed a questionnaire on personality traits and mental distress (response = 69%). Respondents also provided information on educational qualifications at age 33, occupational levels at age 50, and the amount of alcohol consumption at 50 years. The analytic sample comprises 6,478 cohort members (50 per cent females) with complete data. Analysis of response bias in the cohort data showed that the achieved adult samples did not differ from their target sample across a number of critical variables (social class, parental education and gender), despite a slight under-representation of the most disadvantaged groups [25]. Bias due to attrition of the sample during childhood has been shown to be minimal [26]–[27].

### Measures

#### 1. Childhood measures

Parental social class at birth was measured by the Registrar General’s measure of social class (RGSC). RGSC is defined according to occupational status and the associated education, prestige or lifestyle [28] and is assessed by the current or last held job. Where the father was absent, the social class (RGSC) of the mother was used. RGSC was coded on a four-point scale: I professional; II managerial\tech; IIIN skilled non-manual; IIIM skilled manual; IV semi-skilled; and V unskilled occupations [29]. Scores were reversed for the following analyses. Childhood cognitive ability tests [30] consisted of 40 verbal and 40 non-verbal items and were administered at school.

#### 2. Adulthood measures

At age 33, participants were asked about their highest academic or vocational qualifications. Responses are coded to the six-point scale of National Vocational Qualifications levels (NVQ) which ranges from ‘none’ to ‘university degree/higher’/equivalent NVQ 5 or 6. Data on current or last occupation held by cohort members at age 50 were coded according to the Registrar General’s Classification of Occupations (RGSC), described above, using a 6-point classification, and scores were reversed to be compatible with parental social class mentioned above. Personality traits were assessed by the 50 questions from the International Personality Item Pool (IPIP) [31]. Responses (5-point, from “Strongly Agree” to “Strongly Disagree”) are summed to provide scores on the ‘Big-Five’ personality traits: Extraversion, Emotionality/Neuroticism, Conscientiousness, Agreeableness, and Intellect/Openness. Alpha was 0.73 for Extraversion, 0.88 for Emotionality, 0.81 for Agreeableness, 0.77 for Conscientiousness, and 0.79 for Intellect in the study. The z scores were used for the study. Psychological distress was assessed at age 50 using Rutter Malaise Inventory [32]. It comprised of 9 items with Yes/No answers. The content included major psychological disorders such as anxiety, depression, and physical exhaustion. The Alpha was 0.80 in the study. The cut-off point was used to create a dichotomised variable. At age 50 cohort members provided information on the amount of their alcohol consumption. Responses were coded to the six-point scale (0 = Never nowadays/Never had an alcoholic drink, 1 = 1 or 2 units, 2 = 3 or 4 units, 3 = 5 or 6 units, 4 = 7–9 units, 5 = 10 or more units). Binge alcohol use is defined as drinking five or more drinks on a typical day. In addition, the frequency of alcohol consumption was also used in the study, with the question “How often do you have an alcoholic drink of any kind?” 0 = never, 1 = only on special occasions, 2 = once a month, 3 = 2 to 3 times a month, 4 = once a week, 5 = 2 to 3 days a week, 6 = on most days.

### Statistical Analyses

To investigate the prevalence of adult binge alcohol use, first we used ANOVA and *T*-test to examine the characteristics of the study population according to the prevalence of binge alcohol use at 50 years. Second, Spearman rank order correlational analysis was carried out on the measures used in the study. Following this, a serial of logistic regression analyses were conducted to investigate the associations between parental social class, childhood intelligence, educational and occupational attainment, personality traits, and psychological distress using adult binge alcohol use as the outcome variable. STATA version 12 was used for these analyses.

## Results

### Descriptive Analysis

Table 1 shows the characteristics of the study population according to the prevalence of binge alcohol use at 50 years. There were significant gender differences in the dose level of alcohol consumption: men tended to have the greater prevalence on binge drinking than women. *T*-test showed that the differences were statistically significant between men and women on the dose level of alcohol use in a typical day (*t* (*df* = 6477) = 35.01, *p*<.001). Men also had higher percentage of binge alcohol use than women (22.0% vs. 9.8%).

**Table 1:** Social and demographic characteristics of the study population and prevalence of binge alcohol use at age 50.

	n	%	Prevalence of binge alcohol use %
*Gender*			
Male	3241	50.0	22.0
Female	3237	50.0	9.8
*Parental social class at birth*			
Unskilled (V)	486	7.5	21.8
Partly skilled (IV)	751	11.6	18.1
Skilled manual (III)	3199	49.4	17.8
Skilled non-manual (III)	709	10.9	12.6
Managerial\tech (II)	995	15.4	11.2
Professional (I)	338	5.2	5.6
*Educational qualifications at age 33*			
No qualifications	499	7.7	27.9
CSE 2–5/equivalent NVQ1	735	11.3	19.3
O Level/equivalent NVQ2	2252	34.8	16.9
A level/equivalent NVQ 3	998	15.4	16.5
Higher qualification/equivalent NVQ4	1042	16.1	12.3
University Degree/equivalent NVQ 5, 6	952	14.7	8.1
*Own current social class at age 50*			
Unskilled (V)	147	2.3	27.2
Partly skilled (IV)	707	10.9	16.0
Skilled manual (III)	1154	17.8	27.4
Skilled non-manual (III)	1337	20.6	12.2
Managerial\tech (II)	2728	42.1	13.0
Professional (I)	405	6.3	11.1

Table 1 also shows consistent patterns of the percentages of binge alcohol use and parental social class and cohort members’ own educational qualifications and current occupational levels: Participants who came from lower social class, who had lower educational qualifications and were from unskilled or manually skilled occupations had higher percentages of binge alcohol use.

Table 2 shows the Spearman rank order correlations between all variables. The demographic variables and social factors (gender, education and occupation) were all significantly associated binge drinking. Of the personality factors it appeared that those who were most common binge drinkers were Disagreeable Extraverts low on Conscientiousness.

**Table 2:** Spearman rank order correlations of alcohol use, childhood intelligence, personality traits, psychological distress, and demographic variables.

	*Variables*	Mean (SD)	1	2	3	4	5	6	7	8	9	10	11	12	13
1.	Binge drinking	.16 (.37)	**_**												
2.	Frequency of alcohol use	2.80 (1.75)	**−.090**	_											
3.	Gender	.50 (.50)	**−.166**	−.167	_										
4.	Parental social class	3.31 (1.23)	**−.098**	.128	−.026	_									
5.	Childhood intelligence	103.7 (13.0)	**−.121**	.152	.067	.261	_								
6.	Educational qualifications	2.65 (1.46)	**−.122**	.189	−.101	.319	.485	_							
7.	Own occupational levels	4.08 (1.22)	**−.105**	.152	−.026	.215	.327	.473	_						
8.	Extraversion	29.44 (6.60)	**.053**	.125	.083	.044	.030	.082	.132	_					
9.	Emotional stability/Neuroticism	28.90 (7.07)	**−.016**	.053	−.134	.029	.091	.086	.071	.204	_				
10.	Agreeableness	36.83 (5.28)	**−.100**	−.039	.415	.043	.110	.073	.100	.369	.053	_			
11.	Conscientiousness	34.00 (5.26)	**−.058**	.013	.106	.019	.039	.067	.078	.143	.177	.274	_		
12.	Intellect/Openness	32.54 (5.17)	**−.048**	.102	−.016	.142	.274	.319	.255	.387	.095	.343	.229	_	
13.	Psychological distress	1.28 (1.73)	**.029**	−.061	.158	−.032	−.065	−.084	−.078	−.146	−.629	.062	−.102	−.048	_

*Note:* Variables were scored such that a higher score indicated being female, a higher score on binge drinking, a higher frequency of alcohol use, a more professional occupation for parents, higher scores on childhood, highest educational qualification, more professional occupation, higher scores on Extraversion, Emotional stability, Agreeableness, Conscientiousness, Openness, and higher scores on psychological distress.

Childhood cognitive ability was positively associated with the frequency of alcohol use, but it was negatively associated with the dose level of alcohol consumption in a typical day (r = .15 and r = −.12 respectively; *p*<.001).

### Regression Analysis

In order to examine the associations between adult excessive alcohol consumption and social and psychological factors in childhood and adulthood, four models were designed using the logistic regression technique with binge drinking as dependent variable. Model 1 examined childhood factors, parental social class and childhood intelligence, in influencing adult binge alcohol use; Model 2 examined social factors in adulthood in influencing the outcome variable; Model 3 examined adult psychological factors in influencing the outcome variable; and Model 4 examined the associations between all the predictive variables in Models 1–3 and the outcome variable. Gender was controlled in all models.

Table 3 shows the results. Model 1 shows that parental social class (from the skilled non-manual upward to professional occupations, compared to unskilled ones) was significantly associated with adult excessive alcohol use, and childhood cognitive ability was significant and negatively associated with adult binge drinking. Thus hypothesis 1) was confirmed; Model 2 shows that social factors educational qualifications (compared to no qualification), but not occupational levels, were significantly associated with adult binge alcohol use. Thus hypotheses 2) was confirmed; Model 3 shows that among the Big-Five factors, Extraversion, Emotional stability, Agreeableness, Intellect/Openness, but not Conscientiousness, were significantly associated with adult binge drinking. Thus hypothesis 3) was partially confirmed, as Conscientiousness was not significantly associated with adult binge drinking. Psychological distress was also a significant predictor of adult binge alcohol use in the model; and finally in Model 4, when all the childhood and adulthood factors were entered into the equation, parental social class, educational qualifications, traits Extraversion and Agreeableness, and psychological distress were all independent predictors of adult binge alcohol use. Childhood intelligence and emotional stability were associated with adult binge drinking at the significant level of *p*<.10. Apart from gender, the strongest predictor was Extraversion (OR 1.4, 95% CI 1.3 to 1.6; *p*<.001), followed by psychological distress (1.3, CI 1.0 to 1.8; *p*<.05). Compared to those who had no qualifications, participants who had a higher educational qualification tended to have a lower prevalence of binge drinking in adulthood. In this model, parental social class remained to have significant influence in adult alcohol use. Thus hypothesis 4) was partially confirmed in that Conscientiousness was not significantly associated with adult binge drinking whilst Agreeableness was an independent predictor of adult excessive alcohol use.

**Table 3:** Odds ratios (95% CI) for binge alcohol use at age 50, according to parental social class, childhood intelligence, educational qualifications, current social class, personality traits, and psychological distress.

	Odds ratio (95% CI)	*p*-value
**Model 1**		
*Childhood factors*		
Gender	0.39 (0.33, 0.46)***	0.000
Parental social class (*unskilled as reference group*)		
Partly skilled	0.79 (0.57, 1.11)	0.178
Skilled manual	0.84 (0.63, 1.11)	0.227
Skilled non-manual	0.57 (0.40, 0.82)**	0.003
Managerial\tech	0.53 (0.38, 0.76)***	0.000
Professional	0.21 (0.11, 0.39)***	0.000
Childhood intelligence	0.76 (0.70, 0.83)***	0.000
**Model 2**		
*Adult social factors*		
Gender	0.40 (0.34, 0.47)***	0.000
Educational qualifications (*no qualification as reference group*)		
CSE 2–5/equivalent NVQ1	0.61 (0.45,0.83)**	0.002
O Level/equivalent NVQ2	0.57 (0.44, 0.74)***	0.000
A level/equivalent NVQ 3	0.46 (0.34, 0.62)***	0.000
Higher qualification/equivalent NVQ4	0.39 (0.39, 0.54)***	0.000
University Degree/equivalent NVQ 5, 6	0.25 (0.17, 0.36)***	0.000
Own social class (*unskilled as reference group*)		
Partly skilled	0.80 (0.49, 1.32)	0.386
Skilled manual	1.11 (0.69, 1.79)	0.666
Skilled non-manual	0.67 (0.41, 1.09)	0.109
Managerial\tech	0.68 (0.42, 1.10)	0.119
Professional	0.57 (0.32, 1.03)^†^	0.063
**Model 3**		
*Adult psychological factors*		
Gender	0.38 (0.32, 0.46)***	0.000
Extraversion	1.45 (1.32, 1.59)***	0.000
Emotional stability/Neuroticism	0.91 (0.84, 1.00)*	0.047
Agreeableness	0.90 (0.82, 0.99)*	0.023
Conscientiousness	0.95 (0.87, 1.03)	0.182
Intellect/Openness	0.80 (0.73, 0.88)***	0.000
Psychological distress	1.44 (1.12, 1.85)**	0.004
**Model 4**		
*All factors in the study*		
Gender	0.43 (0.35, 0.53)***	0.000
Parental social class (*unskilled as reference group*)		
Partly skilled	0.86 (0.80, 0.93)***	0.397
Skilled manual	0.95 (0.70, 1.28)	0.735
Skilled non-manual	0.65 (0.44, 0.96)*	0.028
Managerial\tech	0.68 (0.47, 0.99)*	0.045
Professional	0.31 (0.16, 0.59)***	0.000
Childhood intelligence	0.91 (0.82, 1.01)^†^	0.068
Educational qualifications (*no qualification as reference group*)		
CSE 2–5/equivalent NVQ1	0.66 (0.47, 0.93)*	0.019
O Level/equivalent NVQ2	0.61 (0.45, 0.83)**	0.002
A level/equivalent NVQ 3	0.53 (0.37, 0.75)***	0.000
Higher qualification/equivalent NVQ4	0.47 (0.33, 0.69)***	0.000
University Degree/equivalent NVQ 5, 6	0.38 (0.25, 0.58)***	0.000
Own social class (*unskilled as reference group*)		
Partly skilled	0.93 (0.53, 1.62)	0.785
Skilled manual	1.28 (0.76, 2.18)	0.356
Skilled non-manual	0.76 (0.44, 1.32)	0.331
Managerial/tech	0.78 (0.46, 1.32)	0.351
Professional	0.69 (0.36, 1.31)	0.252
Extraversion	1.40 (1.27, 1.55)***	0.000
Emotional stability/Neuroticism	0.91 (0.83, 1.00)^†^	0.057
Agreeableness	0.89 (0.81, 0.99)*	0.027
Conscientiousness	0.94 (0.86, 1.03)	0.157
Intellect/Openness	0.97 (0.87, 1.07)	0.533
Psychological distress	1.34 (1.03, 1.75)*	0.029

*Note:* ^†^ *p*<.10;

* *p*<.05;

** *p*<.01;

*** *p*<.001.

## Discussion

This study set out to investigate social and psychological factors in childhood and adulthood which influence adult excessive alcohol consumption. Using a large, nationally representative perspective data in Britain, results showed that both social and psychological factors, in both childhood and adulthood, were significantly and independently associated with binge alcohol use in adulthood. Less Agreeable, intelligent, and educated male Extraverts with lower socio-economic status were most likely to be middle-aged binge drinkers.

This study has confirmed and extended findings of previous studies in the area. It shows that childhood intelligence is significantly associated with the frequency of alcohol consumption as found in previous studies [5]–[7]. This study also demonstrated that intelligence, at least as assessed in childhood, is significantly and negatively associated with potentially hazardous adult binge drinking; that is intelligent children are less likely to have excessive alcohol intake in a given session in adulthood, although they tend to drink alcohol more frequently. There is a distinction between *frequency* and *intensity* of alcohol consumption: the former may incur little harm, or may even have benefit effect on health if it is taken with modest doses [1], whereas the latter, if chronic, may lead to serious health conditions. This finding, however, is in line with the previous findings that intelligent people take better care of themselves, and avoid harmful conditions including certain social leisure settings [8]–[10].

This study also shows that binge drinking is more common in those high in Extraversion, but low in Agreeableness, Openness, Conscientiousness, and Neuroticism. However, after taking account of childhood factors and social factors in adulthood, only Extraversion and Agreeableness remained to be significant predictors of the outcome variable. Whilst it is understandable that Extraverts tend to engage in more social activities that may involve alcohol, their impulsivity and proneness to disinhibitiveness could easily lead to rapid and excessive alcohol consumption, which is a characteristic of binge drinkers. Equally those low in Agreeableness (low on modesty, compliance, generosity, sympathy, and consideration for others) may find it easier to ignore the social disapproval that comes with binge drinking. This also confirms various small scale studies in the area using different populations and different measures of personality [17]–[18].

It is less understandable why Conscientiousness was not independently associated with adult binge alcohol use, as it is often found to be one of the protective factors of physical health and health-related behaviours [11], [13]. One of the explanations could be that Conscientiousness and occupational levels co-vary; indeed among the Big-Five traits Conscientiousness is the strongest predictor of occupational prestige [20].

Parental social class significantly and independently associated with binge alcohol use in adulthood. Thus individuals who came from higher parental social class tend to form more healthy behaviours such as moderate alcohol consumption; conversely individuals who are from lower parental social class with adverse socioeconomic conditions tend to acquire less healthy behaviours, such as alcohol abuse.

Education is important in reducing binge alcohol use for youth [20]. This factor is also important among adults as found in the current study. Findings from scientific investigations are transferred to individuals through various means, however school education allows vast, systematically packed knowledge to be obtained in a relatively short but intense period, together with tried learning methods which may have beneficial effects after one has left school. Thus, it is not surprising that educational achievement, measured by levels of qualifications obtained, is an independent predictor of the amount of adult alcohol consumption, taking into account other confounding factors.

The link between psychological distress and alcohol abuse is well known [22]. Alcohol abuse is more severe among individuals with mental health problems [4]. For those individuals who are less mentally healthy, alcohol may be used initially to reduce anxiety. However, excessive alcohol consumption can lead to increased anxiety and depression, which in turn, leads to greater alcohol abuse [23].

Further studies are required to investigate factors influencing adult binge drinking, as well as differentiating the frequency and dose level of alcohol consumption and related variables.
